# Knowledge, perception, and practical understanding of food labels: A cross‐sectional study among Bangladeshi consumers

**DOI:** 10.1002/fsn3.4366

**Published:** 2024-07-29

**Authors:** Md. Nazrul Islam, Nitai Roy, Felix Kwashie Madilo, Adenike Akinsemolu, Md. Arifuzzaman, Md. Imran Hossain Shakil, Jannatul Ferdous Nishi, Sharmin Akter, Md. Elius, Md. Shahidul Islam

**Affiliations:** ^1^ Department of Post‐Harvest Technology and Marketing, Faculty of Nutrition and Food Science Patuakhali Science and Technology University Dumki Patuakhali Bangladesh; ^2^ Department of Biochemistry and Food Analysis, Faculty of Nutrition and Food Science Patuakhali Science and Technology University Dumki Patuakhali Bangladesh; ^3^ Department of Food Science and Technology, Faculty of Applied Science and Technology Ho Technical University Volta Region Ho Ghana; ^4^ Institute of Advanced Studies University of Birmingham Birmingham UK; ^5^ Department of Botany University of Rajshahi Rajshahi Bangladesh; ^6^ Faculty of Nutrition and Food Science Patuakhali Science and Technology University Dumki Patuakhali Bangladesh; ^7^ Department of Zoology National University Gazipur Bangladesh; ^8^ Department of Economics Hajee Mohammad Danesh Science & Technology University Dinajpur Bangladesh

**Keywords:** Bangladesh, consumer education, expiration dates, food labels, packaged foods

## Abstract

This research aimed to assess consumers' knowledge, perception, and practical understanding of food labels. A validated, structured questionnaire was employed for data collection. Data were collected from 1238 respondents covering all eight administrative divisions of Bangladesh using a nonrandomized convenience sampling method. Linear regression analyses were conducted to establish the relationship between demographic attributes and respondents' practical understanding of food labels. The majority of participants (52.5%) actively read food labels when purchasing a product for the first time. Food labels are regarded as “very important” by 56.2% of respondents and “important” by 35.7% of respondents. Label information is prioritized to highlight the importance of clear production and expiration dates (70%), followed by nutritional composition (56.7%) and source of raw material (52.5%). Despite this, over half of the participants demonstrated a limited practical understanding of the nutritional components on labels. The results of our linear regression analysis suggest that individuals within the age range of 18–28, belonging to Muslim communities, residing in the Dhaka and Khulna divisions, being higher educated, possessing a good understanding of nutrition, and being acquainted with the food safety agency of Bangladesh tend to exhibit a greater degree of practical knowledge regarding food labeling when compared to their counterparts. The study emphasizes the importance of clearer label information, particularly for nutritional value, and calls for targeted educational programs to improve consumer understanding of food labels, with a focus on older age groups and expanded educational efforts.

## Graphical abstract

This study found that 56.2% of consumers consider food labels “very important” and 70% prioritize clear production and expiration dates. However, over half have a limited understanding of nutritional components, indicating the need for clearer label information and educational programs.

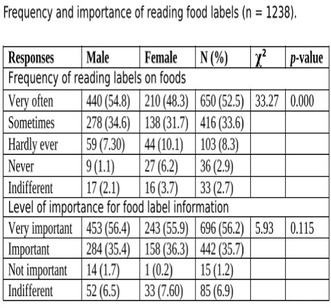

## 1 INTRODUCTION

Every year, noncommunicable diseases (NCDs) cause damage to the lives of about 41 million individuals, primarily in countries with poor financial conditions (WHO, 2018). The relationship between dietary patterns and mortality risk is demonstrated by a 2019 study that found dietary factors to be responsible for 14.6% of deaths for women and 13.5% of deaths for men worldwide (IHME, 2019; Murray et al., 2020). This serious condition affects around 18% of NCD‐related deaths, resulting in an annual economic loss for low‐ and middle‐income countries (LMICs) of approximately US$500 billion, or 4% of their total GDP (Bloom et al., 2011; IHME, 2019; Murray et al., 2020). NCDs have notably influenced mortality rates in Bangladesh. In 2016, these diseases were responsible for 572,600 deaths, accounting for 67% of all deaths that year (WHO, 2020). The prevalence of NCDs has risen significantly over recent decades, a trend expected to persist as Bangladesh undergoes a rapid epidemiological transition (Barua et al., 2021; Chowdhury et al., 2020; Chowdhury, Anik, et al., 2018; Chowdhury, Haque, et al., 2018). In 2000, NCDs accounted for 43% of deaths in Bangladesh, increasing to 59% in 2010 and 70% in 2019 (WB, 2020). This is higher than the average for LMICs (64%) and neighboring countries like India (66%), Nepal (66%), and Pakistan (60%) (Zaman et al., 2015). Among the 20 leading causes of death in 2019, 14 were noncommunicable diseases, while two were communicable (Islam et al., 2023). The rise in these diseases is due to various behavioral, metabolic, and environmental factors. By 2019, all five metabolic risk factors, including dietary factors, were among the top ten risk factors linked to age‐standardized all‐cause deaths, indicating their increased significance over time (Islam et al., 2023).

The rise in noncommunicable diseases (NCDs) and related deaths is significantly influenced by the increased consumption of inexpensive, high‐energy, and low‐nutrient foods, which are frequently referred to as ultra‐processed foods. These foods typically contain high levels of sugar, salt, and/or saturated fat (Mandle et al., 2015). In response to the increasing burden of NCDs, relevant stakeholders are establishing a supportive food environment through policy interventions to encourage the food industry to improve the nutritional quality of their products (Rayner et al., 2013). Effective food labeling can assist consumers in making informed choices, thereby reducing the consumption of energy‐dense and nutrient‐poor foods that contribute to obesity and diet‐related NCDs.

A food label is a graphic depiction of text, print, or another medium on or next to a food product that has several uses. Food labels are essential for enabling appropriate disposal in addition to improving its marketability (Hawkes & World Health Organization, 2004). Nutrition labeling, a subcategory, provides detailed information about a product's nutritional composition (Hawkes & World Health Organization, 2004). In 2013, the Codex Alimentarius, a collaborative commission established by the WHO and FAO, introduced mandatory food labeling to encourage informed and healthy food choices (FAO & WHO, 2019; Godefroy, 2014). Of the 188 countries that formally signed it, about 70 introduced mandatory laws, mainly in LMICs. To protect consumer welfare and encourage healthy eating choices, these regulations usually require food products to prominently display critical information, such as manufacturing and expiration dates, a list of ingredients, suggested serving sizes, and nutritional claims (European Commission, 2016).

Consumer perspectives on food labels vary across geographical and cultural contexts. European consumers generally understand nutritional information and health behaviors, but their interpretation of label information is less precise and accurate (Gregori et al., 2014). The study reveals that nutrition‐related diseases are an important issue that unfortunately receives inadequate attention from governments and the media. Consumers were confused about nutrition labeling, particularly regarding technical and numerical information, such as serving sizes and their relationship with energy and calories. The survey highlights the need for more comprehensive and accurate information on nutrition‐related diseases. Food labels in the United States and Canada give consumers critical information regarding the quantity, nutritional profile, and composition of food, enabling them to make well‐informed purchasing decisions (Wingfield, 2016). In South Africa, health information on food labels was generally well received, though some expressed a lack of interest, time constraints, price concerns, and a tendency to habitually purchase without reading food labels (Bosman et al., 2014). Indian customers believe every food label should include the product price, manufacturing date, best before and expiry dates, name and location of the product producer, and nutritional content information for better health risk management (Ali & Kapoor, 2009). Similarly, a recent study conducted in Bangladesh, specifically in the Lalmonirhat District, emphasizes the significance of accurate and informative labeling in meeting customer expectations and ensuring satisfaction (Shahiduzzaman & Naskar, 2022). Alarmingly, only 44.4% of individuals take the time to read labels, indicating significant gaps and inconsistencies in consumer behavior and attitudes toward packaged food products. This underscores the urgent need for further research to comprehensively address these concerns (Shahiduzzaman & Naskar, 2022).

In Bangladesh, there are specific laws that dictate the criteria for labeling: (i) The Packaged Food Labeling Regulations of 2017, (ii) Bangladesh Standards of Weights and Measures (Commodity Packing) Rules, 2007, (iii) Bangladesh Import Orders from 2015 to 2018, and The Food Safety Act of 2013 (Chopra & Bareja, 2022). Bangladesh also has various organizations that play vital roles in ensuring the quality and integrity of food items and labeling, including the Bangladesh Standards and Testing Institution (BSTI) and the Bangladesh Food Safety Authority (BFSA). The Bangladesh Packaged Food Labelling Act 2017 mandates clear and concise label information, including product price, manufacturing date, best before date, and expiration date. Labels must also include the name and address of the manufacturer, packager, supplier, or marketer, health risk warnings, dietary content information, and quantitative data on artificial components. Additionally, labels should provide net weight, volume, safety precautions, nutritional content, food additives, storage instructions, and ethical information.

Unfortunately, enforcement of these regulations is weak in Bangladesh. Many products do not fully comply with labeling rules, indicating a lack of thorough control (Ali & Shahnewaj., 2017). The BSTI faces challenges in effectively enforcing food safety standards due to the absence of well‐defined methods for determining expiry dates and clear terminology. The BSTI only verifies the inclusion of production and expiry dates on labels without considering the correlation between the date and the food's safety and nutritional value. Moreover, BSTI does not monitor date labeling as part of its food safety program and admits it lacks the means to verify incorrect or fraudulent date labeling (Ali & Shahnewaj., 2017). Small‐scale manufacturers sometimes resort to misleading date labeling practices to enhance their business image. Moreover, the Consumer Association of Bangladesh (CAB) implements consumer education initiatives emphasizing the importance of avoiding expired food but does not advocate for the government to standardize date labeling terminology or ensure consumer awareness to eliminate labeling confusion (Ali & Shahnewaj., 2017).

In a survey, 33 different companies' brands of biscuits, both locally and imported, were examined by the prestigious Consumers Association of Bangladesh (CAB) (CAB, 2023). The results show a worrying picture: 76% of the products were not properly labeled or did not have the certification markings required by the BSTI. Moreover, a significant proportion, almost 86%, do not provide an expiration date, which is an essential piece of information for customers. The fact that 83% of the products omit to state their weight raises concerns regarding customer understanding and transparency, which is equally concerning. When the CAB expanded the poll to include packaged jams and jellies, troubling results were obtained. An important detail for customers with dietary preferences or limits is the ingredients list, which in 13.7% of the brands were found to be missing. Furthermore, 23.5% failed to include the dates of manufacturing and expiration, which are essential for guaranteeing the safety and freshness of the goods. The retail price was missing from the label in an astounding 54.9% of cases, depriving customers of crucial information needed to make informed decisions. Furthermore, it is worth noting that a comparable proportion (83%) of products are being sold at elevated prices by sellers, which has the potential to undermine consumer trust (CAB, 2023).

The severity of the situation is further highlighted by statistics that show that 40% of our Bangladeshi products are rejected in the EU and America due to faulty labeling, and that allergens that are not reported are responsible for about 30% of recalls (SGS Bangladesh, 2021). This insufficiency is a serious problem, particularly in Bangladesh, where the regulatory authorities have very little ability to enforce mandatory food standards—just 12% of total food requirements are considered mandatory (The New Age, 2021).

In light of the urgency of these issues and the need to protect consumer health, the purpose of our study is to evaluate Bangladeshi consumers' knowledge, perception, and practical understanding of food label information. Additionally, we want to investigate how these variables relate to different demographic traits to shed insight into the nuances of Bangladeshi consumers' ability to understand food labels. This study aims to address the particular difficulties faced by consumers in the Bangladeshi setting while also offering significant insights into the worldwide conversation on food labeling. We hope that the information we gather will empower consumers and inform stakeholders and policymakers alike, resulting in a more transparent and health‐conscious food industry in Bangladesh and beyond.

## 2 MATERIALS AND METHODS

### 2.1 Study setting description

The study utilized a cross‐sectional approach in Bangladesh, located in the north‐eastern region of South Asia. As reported in 2022, the population of Bangladesh stands at an estimated 165.1 million, representing about 2.11% of the worldwide population (BBS, 2022). The age distribution indicates a youthful demographic, with a median age of 27.6 years. Urban dwellers make up 37.4% of the total population, and with a density of 1119 individuals per square kilometer, a significant 91% of the populace identifies as Muslim.

### 2.2 Sample size and participants

A nonrandom convenience sampling method was applied for recruiting participants. Sample size estimation leveraged the Openpi online software, accessible at https://www.openpi.com/SampleSize/SSPropr.htm. The Bangladesh Bureau of Statistics (BBS) reported 165.1 million of the Bangladeshi population in 2022 (BBS, 2022). Subsequent statistical power analysis for sample size was based on this figure. The sample size was determined using the “EPI INFO” version 7.4.2.0 software provided by the Centers for Disease Control and Prevention (CDC) in the United States. The estimated sample size calculated was 1088, with a confidence interval of 99.9%, an expected frequency of 50%, a margin of error of 5%, and a design impact of 1.0. A total of 1382 participants were initially recruited. However, the final sample comprised 1238 (89.58%) participants spread across the eight administrative divisions. Participants in the study were Bangladeshi citizens. Participants had to meet certain requirements to be eligible, including being at least fourteen years old, being of any gender, being fluent in Bengali or English, and residing in their respective divisions for at least 1 year. Participants were excluded if they were nonresidents of Bangladesh, under the age of 14, unable to understand Bengali or English, or had not resided in their respective divisions for a minimum of 1 year.

### 2.3 Design and administration of the questionnaire

To gauge the Bangladeshi consumers' understanding, usage, and comprehension of food labeling, a self‐administered questionnaire was formulated. This tool sourced questions from validated instruments, including those by Madilo et al. (2020), Gomes et al. (2020), and Ponnudurai et al. (2019). Given the unique dietary habits in Bangladesh, several survey questions underwent necessary modifications. Initially crafted in English, a language expert translated the survey into Bengali. The questionnaire was then back‐translated by a separate translator to ensure consistency and accuracy of the translations. Preliminary validation was sought through a pilot study involving 45 consumers, assessing aspects like clarity, readability, and completion time. Feedback from this pilot led to revisions, although its results were not incorporated into the primary survey.

The questionnaire had three sections. The first section collected demographic details, such as gender, age, religion, marital status, place of residence (rural and urban), monthly family income (BDT), having children, education level, occupation, administrative divisions of Bangladesh, daily calorie intake, health perceptions, diet adherence, self‐reported nutrition knowledge, and familiarity with food safety agencies. Similar to the previous study (Roy et al., 2023), there were three groups for monthly family income: less than 15,000 Bangladeshi Taka (BDT), between 15,000 and 30,000 BDT, and over 30,000 BDT (110.16 BDT = 1 USD). For religion, others represent those studies belonging to a religion other than Muslim and Hindu, that is, Christianity, Buddhism, etc. The participant's health perception was categorized as poor, fair, good, very good, or excellent, indicating their overall well‐being and frequency of health issues. Self‐reported nutritional knowledge describes different levels of knowledge regarding nutrition, ranging from basic understanding to good to excellent knowledge, with varying levels of confidence in dietary choices (see Table 1 for further details).

**TABLE 1:** Different sections of the questionnaire and their corresponding study objectives.

Section	Subsection	Study objective	Description
Section 1	Demographic characteristics	To understand the demographic profile of the participants	Collected details such as gender, age, religion, marital status, place of residence (rural and urban), monthly family income (BDT), having children, education level, occupation, administrative divisions of Bangladesh, daily calorie intake, health perceptions, diet adherence, self‐reported nutrition knowledge, and familiarity with food safety agencies. Income categories: less than 15,000 BDT, 15,000–30,000 BDT, over 30,000 BDT
Section 2	To what extent do you consider food labels to be important?	To determine the perceived importance of food labels among consumers	This section investigates various aspects of consumers' interactions with food labels. It examines the frequency with which consumers read labels when purchasing products for the first time, their perceived importance of food labels, and the specific information they deem crucial, such as expiry dates, nutritional details, and country of origin. The study also explores consumers' desires for additional information on labels, preferences for more or improved label content (including details like raw materials, nutrient contents, and production details), and their satisfaction with the adequacy of current label information. Additionally, the research identifies sources beyond labels that consumers use to gather information about food products and assesses their agreement with statements regarding the adequacy and clarity of food label information
	Indicate the level of importance you attach to the label information provided below	To identify which specific information on food labels consumers consider important	
	What information do you want to see on the food labels before you buy?	To understand what additional information consumers want to see on food labels	
	When I read food labels what more or better information do I wish to see?	To gauge consumers' desire for more or improved information on food labels	
	To what degree do you get the information you need on the labels in order to buy foods of your choice?	To assess whether consumers feel they get enough information from food labels	
	From which other sources do you get information on food you purchase?	To identify other sources consumers use to get information about food products	
	How much do you agree with the following statements about the information on food labels of packaged foods?	To determine consumers' agreement with various statements about the adequacy and clarity of food labels	
Section 3	Practical understanding of nutritional information	To evaluate participants' practical understanding of nutritional information on food labels	Assessed practical understanding of nutritional labeling through a comparison of two products' nutritional facts. Included are questions on energy levels, impact on cholesterol, mineral content, and sugar content, as well as identifying the most and least important nutritional information on labels

The second segment explored consumers' knowledge and perceptions of food labels. To assess knowledge and perception, we conducted an evaluation that included inquiries about their frequency of reading labels on newly purchased food items (with response choices ranging from “Very often” to “Indifferent”) and the level of importance they place on food labels (response options included “Very important,” “Important,” “Not important,” and “Indifferent”). We asked them about their expectations for label contents and how important different types of label information are to them, such as expiry date, nutritional details, and country of origin. We also asked them what specific information they wanted to see on food labels before making a purchase. We also evaluated their interest in obtaining more comprehensive information regarding raw materials and nutrient contents. Finally, we offered various options for individuals to determine their main sources of food label information, including academic studies, the internet and social media, friends and neighbors, and healthcare professionals such as doctors, dietitians, and pharmacists.

The concluding section evaluated participants' hands‐on knowledge of nutritional information on food labels. To determine the participants' practical understanding of nutritional labeling, we set the nutritional facts of two products (chocolate cookies/product A and chip cookies/product B) and asked them seven questions (e.g., Which of the two products gives you a higher energy level if the right quantity is consumed at a given time?). Participants were asked to provide an open‐ended response regarding their anticipated frequency of consuming product A. Respondents were given the choice between options A and B to compare two products based on criteria such as energy level, impact on cholesterol, mineral content for bone health, and sugar content per 100 g. In addition, participants were asked to select the most and least important nutritional information on food labels. They could choose from options such as calories/energy, total fats, trans fats, saturated fats, sugars, vitamins, and minerals. A higher score in this case denotes a more practical comprehension of food labeling.

### 2.4 Data collection

In the span between September and November 2022, senior students specializing in nutrition and food science from Patuakhali Science and Technology University were enlisted and trained to gather data through a structured, self‐administered questionnaire. This training was spearheaded by a food science expert and the lead researcher, emphasizing effective data collection techniques and a comprehensive understanding of the study's objectives. Respondents were sourced from every division in Bangladesh, applying convenience sampling. Both rural and urban locales—including educational institutions, supermarkets, malls, and residential areas—were integral to the data collection process. Given the literacy challenges prevalent in rural sectors, interviewers verbally communicated the questions and potential responses to participants, assisting them in questionnaire completion. In urban environments like shopping centers, participants were prompted to independently fill out and return the questionnaire to the interviewer within a 30‐min window. Each respondent was informed about the study's aim, assured of data confidentiality, and presented with a consent form. Interviewers stepped in to clarify and explain any sections that participants found challenging, ensuring comprehension. While the questionnaire was intended to be self‐administered, the investigator had to complete a few questionnaires for respondents who lacked formal education. The questionnaire, on average, took about 15 min to complete.

### 2.5 Ethical considerations

Prior to initiating the study, ethical approval was obtained from the Institutional Ethical Committee (IEC) of the Patuakhali Science and Technology University (PSTU), Bangladesh. The committee meticulously reviewed and subsequently approved the study's methodology (Approval no: PSTU/IEC/2022/38). Key ethical protocols encompassed transparently briefing participants about the research's significance, objectives, and methodologies. Further, participants were unequivocally assured that their data and responses would be safeguarded with the utmost anonymity. The emphasis was laid on the fact that findings derived from this study would be exclusively earmarked for academic dissemination.

### 2.6 Data analysis

The data were subsequently analyzed using Statistical Package for the Social Sciences (SPSS) version 27.0. Preliminary descriptive statistics were employed to outline the demographic and other pertinent features (e.g., frequencies, percentages, and means). Practical knowledge was then calculated by assigning a score of 1 for each correct answer and 0 for incorrect responses, yielding a potential score range between 0 and 7 for this section. The Kruskal–Wallis *H* and Mann–Whitney *U* tests were implemented to discern mean value discrepancies. The Kruskal–Wallis *H* test was employed to compare the scores of three or more independent groups, such as those with varying income levels or educational levels. To compare the differences between two independent groups, such as rural and urban residents, the Mann–Whitney *U* test was implemented. To check for multicollinearity, GVIF and tolerance were calculated for the final section of the questionnaire (which included seven questions), with a GVIF threshold value of 10. No multicollinearity was detected, deeming all the independent variables fit for linear regression. After model fitting, all assumptions underpinning linear regression were scrutinized. All statistical tests were two‐tailed and conducted at a 95% confidence level, considering results significant when the *p*‐value was below .05.

## 3 RESULTS AND DISCUSSION

### 3.1 Characteristics of the study participants

The characteristics of the respondents are delineated in Table 2. The data reflect a balanced gender distribution, with males comprising 64.9% and females accounting for 35.1% of the sample. Notably, a significant portion of the respondents, 59.9%, fell within the 19–28 age bracket. When examining the economic aspect, approximately 58% of respondents disclosed a monthly family income ranging from 15,000 to 30,000 taka (1 USD = 117.56 Bangladeshi taka). The religious breakdown showed a predominant Muslim representation at 84.6%. Marital status varied among participants; however, a majority (55.6%) identified as single, while roughly 43.1% were married. In terms of regional distribution, both urban (44.7%) and rural (55.3%) respondents contributed to the study. Geographically, participants hailed from every division in Bangladesh, with the Dhaka division being the most represented at 16.6%, while the Chittagong division had the least at 8.9%. An encouraging trend emerged regarding educational qualifications, as the majority (43.1%) held advanced degrees, either at the B.Sc./Masters level or above. Pertinent to health and dietary habits, nearly half of the respondents (48.5%) perceived their health status as fair, and a significant 77.2% did not adhere to any specific dietary regimen.

**TABLE 2:** General characteristics of the study participants (*n* = 1238).

Characteristics	Categories	Total (*N*)	Percentage
Age groups (28.82 ± 10.898)	18 or below	69	5.6
	19 to 28	742	59.9
	29 to 38	228	18.4
	39 to 48	113	9.1
	Above 48	86	6.9
Gender	Male	803	64.9
	Female	435	35.1
Monthly family income (BDT)	Below 15,000	196	15.8
	15,000 to 30,000	715	57.8
	Above 30,000	327	26.4
Religion	Muslim	1047	84.6
	Hindu	181	14.6
	Others^a^	10	0.8
Marital status	Married	533	43.1
	Unmarried	687	55.5
	Widowed/Divorced	18	1.5
Having children	Yes	457	36.9
	No	781	63.1
Place of residence	Urban	554	44.7
	Rural	684	55.3
Administrative division	Barishal	141	11.4
	Chattogram	110	8.9
	Dhaka	206	16.6
	Khulna	154	12.4
	Mymensingh	150	12.1
	Rajshahi	164	13.2
	Rangpur	187	15.1
	Sylhet	126	10.2
Education of participants	No formal education	53	4.3
	Primary	61	4.9
	Secondary	192	15.5
	Higher Secondary	399	32.2
	B.Sc./Masters or above	533	43.1
Occupations	Student	577	46.6
	Housewife	202	16.3
	Employment	221	17.9
	Job/Service	64	5.2
	Business	159	12.8
	Retired or pensioner	15	1.2
Health perception	Poor	89	7.2
	Fair	601	48.5
	Good	439	35.5
	Very good	83	6.7
	Excellent	26	2.1
Follow the diet chart	Yes	282	22.8
	No	956	77.2
Self‐reported nutrition knowledge	Not knowledgeable	172	13.9
	Somewhat Knowledgeable	865	69.9
	Knowledgeable	201	16.2
Calculate daily calorie intake	Never	636	51.4
	Sometimes	520	42.0
	Most of the time	82	6.6
Idea about the food regulatory agencies of Bangladesh	Yes	714	57.7
	No	524	42.3

Abbreviations: BDT, Bangladeshi Taka; 110.16 BDT, 1 USD.

^a^ Buddhist, Christians.

### 3.2 Food label information: utilization and significance

In exploring consumer behaviors toward packaged food labels, a majority of the participants, 52.5%, reported regularly reading labels when purchasing a product for the first time (Table 3). A smaller group, 33.6%, indicated they occasionally perused labels, while only 2.9% stated they never did. The observed patterns indicate that participants are actively involved in examining food labels, which aligns with the findings of the study conducted in Brazil by Sekiyama et al. (2019). While cultural differences may exist, this consistency suggests a potential universality in consumer behavior when encountering new products. However, the findings presented by previous studies contrasted with this engagement (Bayram & Ozturkcan, 2022; Madilo et al., 2020; Sajdakowska et al., 2022). The disparity with other studies underscores the well‐known fact that consumer behaviors and perceptions differ between studies or communities. This emphasizes the need to consider context when interpreting results and making decisions, rather than a need for more investigation.

**TABLE 3:** Frequency and importance of reading labels (*n* = 1238).

Responses	Male	Female	*N* (%)	χ^2^	*p*‐value
Frequency of reading labels on foods					
Very often	440 (54.8)	210 (48.3)	650 (52.5)	33.27	.000
Sometimes	278 (34.6)	138 (31.7)	416 (33.6)		
Hardly ever	59 (7.30)	44 (10.1)	103 (8.3)		
Never	9 (1.1)	27 (6.2)	36 (2.9)		
Indifferent	17 (2.1)	16 (3.7)	33 (2.7)		
Level of importance for food label information					
Very important	453 (56.4)	243 (55.9)	696 (56.2)	5.93	.115
Important	284 (35.4)	158 (36.3)	442 (35.7)		
Not important	14 (1.7)	1 (0.2)	15 (1.2)		
Indifferent	52 (6.5)	33 (7.60)	85 (6.9)		

When delving into the perceived importance of food labels, a notable 56.2% of participants deemed them “very important” (Table 3). Another 35.7% marked them as “important,” while a mere 6.9% remained “indifferent” and 1.2% rated them as “not important.” These results underline the considerable value respondents place on food labeling. Such valuations are reinforced by Bandara et al. (2016), who posited that the information on a packaged food label profoundly influences consumer purchasing decisions (Bandara et al., 2016). Additionally, Ballco et al. (2019) argued that well‐structured food labels can address issues like malnutrition, poor dietary habits, diabetes, and obesity, emphasizing the importance of providing accurate product information to guide consumers (Tonkin et al., 2016). This implies that accurate and thorough information on food labels is beneficial for both public health programs and individual consumers.

**FIGURE 1:**
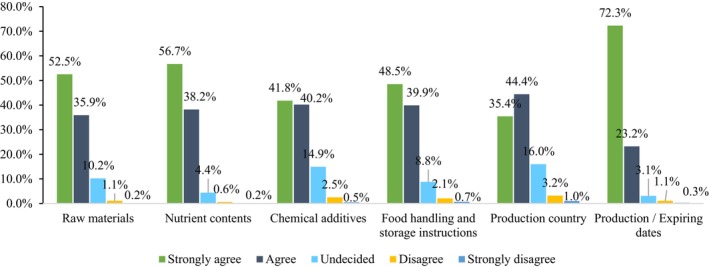
Important elements that need more information on the food labels.

Diving deeper into label specifics, when participants were asked to prioritize the information they deemed crucial on food labels, a strong majority—over 70% of respondents—strongly agreed on the need for clear “production and expiry dates” indicating a robust need for openness regarding the product's freshness and shelf life (Figure 1). This was followed by “nutrient composition” (56.7%), the “source of raw materials” (52.5%), “food handling and storage instructions” (48.5%), and lastly, “production countries” (35.4%) (Figure 1). Several studies corroborate these preferences, highlighting the importance of expiry dates (Ahuja et al., 2019; De‐Magistris et al., 2017), nutritional composition (Deliza et al., 2020; Nobrega et al., 2020), and product pricing (Hellier et al., 2012). Most tellingly, not a single respondent (0.2%) dismissed the significance of detailed nutritional information on food labels, a sentiment that aligns seamlessly with the conclusions of Sajdakowska et al. (2022). This consensus shows that the majority of consumers understand how important it is to have access to nutritional information when making decisions about what to eat.

### 3.3 Assessment of food label information in packaged goods in Bangladesh

**FIGURE 2:**
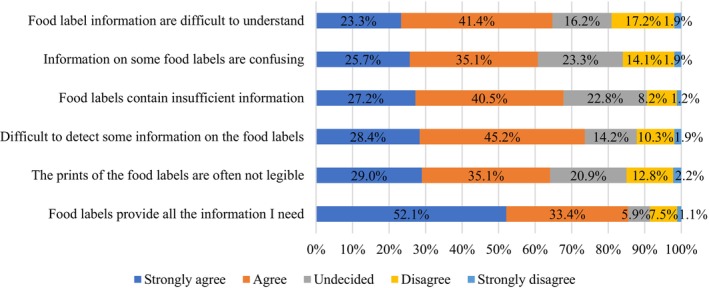
Assessment of labels on packaged food purchased in Bangladesh.

Figure 2 showcases respondents' perceptions of the adequacy of food label information on packaged foods purchased within Bangladesh. The majority, over 50%, strongly concurred that the packaged foods they acquired generally offered satisfactory label information. In contrast, less than 10% expressed dissent, either disagreeing or strongly disagreeing with the notion. However, a significant proportion, surpassing 40%, agreed that certain label details are either hard to discern, challenging to comprehend, or simply lacking in sufficient clarity.

In a study conducted by Hudson and Hartwell (2002), participants expressed that the text size was frequently inadequate and posed challenges for individuals with tunnel vision and visual impairments (Hudson & Hartwell, 2002). In the present study, over 60% of respondents also agreed strongly with this finding. Furthermore, additional studies have revealed significant issues with labels, such as a lack of comprehension, ambiguity, inadequate font size, inadequate accuracy of information (Cowburn & Stockley, 2005), a lack of trustworthiness (Van der Merwe & Venter, 2010), and inadequate information accompanying health claims (Svederberg & Wendin, 2011). Additional investigations corroborated the findings of Van der Merwe et al. (2014), showing that the majority of respondents (54%) who expressed significant agreement regarding the difficulties also concurred regarding the perplexity induced by the content. Consumers encountered certain technical and numerical information that was perplexing (Cowburn & Stockley, 2005) and challenging to comprehend (Svederberg & Wendin, 2011). However, it is worth noting that confusion may also arise from an excessive amount of information (Pomeranz, 2011). Based on our findings, we propose increasing the font size to improve readability and simplifying content to reduce perplexity. To avoid information overload, we recommend prioritizing essential details on labels and providing additional information through digital means.

### 3.4 Prioritization of food label information by consumers in Bangladesh

**FIGURE 3:**
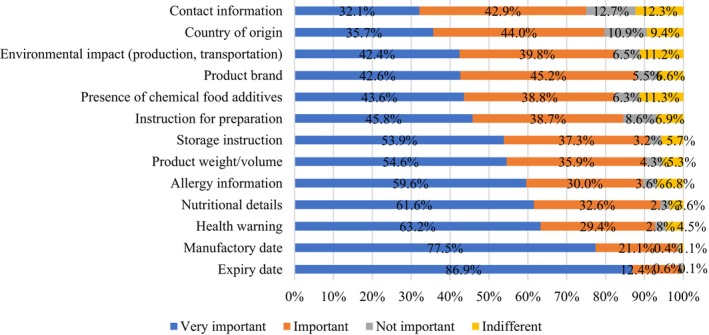
The level of importance attached to the food label information.

In assessing the weightage participants placed on various elements of food label information on packaged goods, a clear hierarchy emerged. An overwhelming majority regarded the expiry date as paramount (86.9%), followed by the manufacturing date (77.5%), health warnings (63.2%), nutritional specifics (61.6%), and finally, allergy information (59.6%) (as represented in Figure 3). Notably, both manufacturing and expiry dates stood as vital indicators of product safety, serving as primary shields against potential foodborne illnesses. Interestingly, minimal respondents classified any of these elements as ‘unimportant,’ hinting at a discerning consumer base that diligently perused food labels. The results revealed an intriguing observation: more respondents placed importance on food safety elements, such as expiration dates and manufacturing dates, compared to nutritional information, when evaluating food labels. Previous research has indicated that in various European populations, consumers place less significance on nutrition information displayed on food labels compared to food safety information. This includes details such as expiration date, freshness, genetic modification, and origin (Grunert & Wills, 2007). The cross‐cultural coherence of consumer priorities underscores a wider pattern, stressing the universal significance that consumers attach to components like manufacturing details and expiration dates.

**FIGURE 4:**
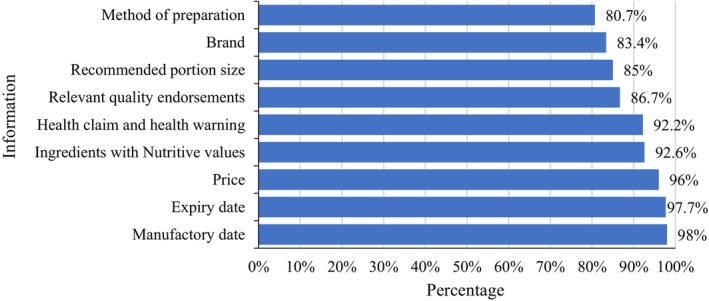
Information one wants to see on the food labels before purchasing.

Additionally, when quizzed about essential label details preceding a purchase, participants prominently featured manufacturing (98%) and expiry dates (97.7%) at the apex (as depicted in Figure 4). Price followed closely (96%), reflecting its significant influence on purchasing decisions. Subsequent in priority were ingredients accompanied by their nutritive profiles (92.6%) and health claims and health warnings (92.2%), while methods of preparation ranked lowest (80.7%). This pattern aligns with findings from Robert and Chandran (2017), who accentuated the predominant consumer emphasis on expiry dates and prices during their packaged food selection process (Robert & Chandran, 2017).

### 3.5 Consumer perception of food label completeness and information sources in Bangladesh

**FIGURE 5:**
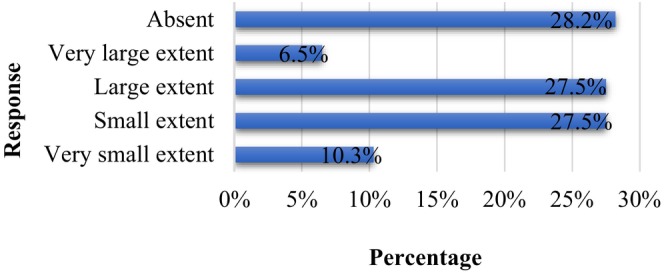
The degree of information consumers' needs on food labels.

**FIGURE 6:**
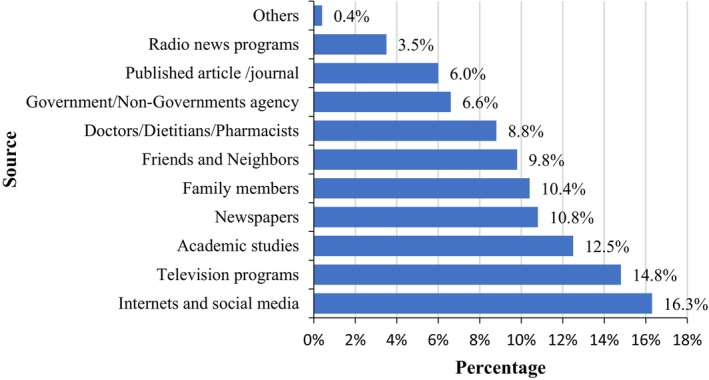
Sources of food label information.

Figure 5 elucidates the degree to which participants obtain food label information. A higher proportion felt that the information they sought was provided to a “small extent,” while a subsequent chunk believed it was provided to a “large extent.” This feedback suggests that manufacturers are making commendable efforts to cater to consumers' informational needs, even though there is always room for improvement. As for the predominant avenues through which consumers garner vital food label details, Figure 6 offers clear insights. A significant proportion identified “internet and social media” as their primary information source (16.3%), followed by television (14.8%), academic studies (12.5%), and newspapers (10.8%). Surprisingly, radio news programs lagged (3.5%), ranking as the least referenced medium. These findings align with the observations from Madilo et al. (2020), emphasizing the evolving and digitized nature of consumer information acquisition in contemporary times.

### 3.6 Understanding practical knowledge of nutritional information on packaged foods in Bangladesh

Table 4 delineates the respondents' practical comprehension of nutritional data on packaged foods available in Bangladesh. Intriguingly, the data suggest a prevailing deficiency in the practical understanding of nutritional labeling. A significant portion, constituting less than 50% of respondents, struggled to interpret the nutritional information accurately. Notably, a mere 11% could aptly discern the frequency with which they should consume a specified product, ‘Product A’. This finding is reminiscent of another study by Robert and Chandran (2017), wherein only 17% of participants indicated their proficiency in accurately gauging nutritional values from food labels. According to Lubman et al. (2012), 55% of participants used food labels; however, only 32% were able to accurately comprehend the information on the labels (Lubman et al., 2012). Furthermore, nearly 30% of respondents incorrectly recognized the product as providing increased energy levels, and a substantial 48.9% of respondents identified a product as having a positive impact on cholesterol levels. These results corroborate those of Madilo et al. (2020). Lubman et al. (2012) reported that 68% and 58% of their sample, respectively, struggled to answer the two questions assessing their proficiency in calculating the total calorie and fat content per package (Lubman et al., 2012). This suggests that many of the participants were unfamiliar with the concept of servings per container.

**TABLE 4:** Respondents' practical nutritional knowledge of food labels (*n* = 1238).

Questions	Wrong answer, *N* (%)	Correct answer, *N* (%)	Do not know, *N* (%)
How many different times are you expected to consume product A?	867 (70.0)	141 (11.4)	230 (18.6)
Which one of the two products gives you a higher energy level if the right quantity is consumed at a given time?	365 (29.5)	585 (47.3)	288 (23.3)
Which of the two products is likely to have a positive impact on the cholesterol level in the blood?	605 (48.9)	272 (22.0)	361 (29.2)
Which of the two products is good for healthy bones and teeth development in terms of mineral content?	320 (25.8)	415 (33.5)	503 (40.6)
Which of the two products will have a higher sugar content per 100 g of the product?	272 (22.0)	569 (46.0)	397 (32.1)
Which of the following nutritional information on food labels do you consider most important?	972 (78.5)	19 (1.5)	247 (20.0)
Which of the following nutritional information on food labels do you consider least important?	776 (62.7)	61 (4.9)	401 (32.4)

The survey findings indicated that 25.8% of respondents provided incorrect responses, while 40.6% expressed uncertainty regarding which of the two products was more beneficial for promoting healthy bones and teeth development based on mineral content. Additionally, 22.0% of participants incorrectly identified, and 32.1% were uncertain about which product would have a higher sugar content per 100 g. In the study conducted by Kreuter et al. (1997) and Cowburn and Stockley (2005), it was found that consumers face challenges when it comes to converting information from “g per 100 g” to “g per serving” and understanding serving size information (Cowburn & Stockley, 2005; Kreuter et al., 1997). Therefore, utilizing verbal descriptions may be more effective in assisting consumers in comparing products and understanding their significance in their overall diet than relying solely on complex numerical data or recommended reference values.

Furthermore, it was found that 78.5% of the participants provided inaccurate responses when asked about the most crucial nutritional details displayed on food labels. Similarly, 62.7% of the participants were mistaken when identifying the least significant information on these labels. The inability to comprehend or apply food label information, particularly nutritional content, can precipitate myriad health challenges. Misunderstanding or disregarding such information can be a precursor to conditions such as obesity, hypertension, goiter, type 2 diabetes, cardiovascular diseases, stroke, osteoporosis, and, in severe cases, even mortality. Hence, it becomes imperative to instigate educational initiatives. Tailored programs should be established across schools, marketplaces, and communities, emphasizing the significance of food label comprehension. Specifically, these platforms should guide deciphering and applying the nutritional data of packaged foods, enabling informed consumer choices.

### 3.7 Demographic influences on the practical knowledge of food labeling

This study sought to discern the potential influence of demographic variables on the practical comprehension of food labeling. The findings, detailed in Table 5, underscore that certain demographic attributes did not significantly sway the respondents' practical knowledge of nutritional labels; these included gender, monthly income, marital status, place of residence, and occupation (*p* > .05).

**TABLE 5:** Linear regression assessing practical knowledge about food labeling.

Characteristics	Categories	Practical food labeling knowledge^a^				
		Unstandardized coefficients (B)	Standardized coefficients (β)	*p*‐value	95% CI	
					LL	UL
	(Constant)	−0.05		.856	−0.62	0.51
Age	18 or below	0.86	0.16	**.000**	0.41	1.31
	19 to 28	0.55	0.22	**.001**	0.22	0.89
	29 to 38	0.29	0.09	.069	−0.02	0.60
	39 to 48	0.15	0.04	.392	−0.19	0.48
	Above 48	Ref.	Ref.	–	Ref.	Ref.
Gender	Male	0.01	0.00	.912	−0.16	0.18
	Female	Ref.	Ref.	–	Ref.	Ref.
Religion	Muslim	0.26	0.08	**.008**	0.07	0.45
	Others^b^	0.14	0.01	.715	−0.59	0.86
	Hindu	Ref.	Ref.	–	Ref.	Ref.
Monthly family income (BDT)	Below 15,000	0.06	0.02	.626	−0.17	0.29
	15,000 to 30,000	0.12	0.05	.136	−0.04	0.28
	Above 30,000	Ref.	Ref.	–	Ref.	Ref.
Marital status	Unmarried	0.08	0.03	.532	−0.17	0.33
	Widowed/Divorced	−0.08	−0.01	.770	−0.64	0.48
	Married	Ref.	Ref.	–	Ref.	Ref.
Having children	Yes	0.01	0.00	.953	−0.25	0.27
	No	Ref.	Ref.	–	Ref.	Ref.
Place of residence	Urban	−0.04	−0.01	.638	−0.18	0.11
	Rural	Ref.	Ref.	–	Ref.	Ref.
Administrative division	Sylhet	0.20	0.05	.167	−0.08	0.48
	Barishal	0.22	0.05	.168	−0.09	0.53
	Chattogram	−0.03	−0.01	.810	−0.31	0.25
	Dhaka	0.44	0.12	**.003**	0.15	0.72
	Khulna	0.51	0.14	**.001**	0.22	0.80
	Mymensingh	−0.34	−0.10	**.017**	−0.63	−0.06
	Rajshahi	0.03	0.01	.823	−0.25	0.31
	Rangpur	Ref.	Ref.	–	Ref.	Ref.
Education of participants	Primary	0.04	0.01	.870	−0.39	0.46
	Secondary	0.29	0.09	.131	−0.09	0.65
	Higher Secondary	0.54	0.21	**.004**	0.17	0.90
	B.Sc./Masters or above	0.52	0.21	**.006**	0.14	0.89
	No Formal Education	Ref.	Ref.	–	Ref.	Ref.
Occupations	Housewife	0.00	0.00	.980	−0.29	0.28
	Employment	0.20	0.06	.099	−0.04	0.44
	Job/Service	0.14	0.03	.387	−0.17	0.44
	Business	0.06	0.02	.654	−0.21	0.33
	Retired or pensioner	0.48	0.04	.158	−0.19	1.15
	Student	Ref.	Ref.	–	Ref.	Ref.
Follow the diet chart	Yes	0.09	0.03	.278	−0.08	0.26
	No	Ref.	Ref.	–	Ref.	Ref.
Self‐reported nutrition knowledge	Somewhat Knowledgeable	0.19	0.07	.082	−0.02	0.40
	Knowledgeable	0.43	0.13	**.002**	0.16	0.70
	Not Knowledgeable	Ref.	Ref.	–	Ref.	Ref.
Calculate daily calorie intake	Sometimes	−0.03	−0.01	.692	−0.18	0.12
	Most of the time	−0.20	−0.04	.178	−0.50	0.09
	Never	Ref.	Ref.	–	Ref.	Ref.
Idea about the food safety agency of Bangladesh	Yes	0.23	0.09	**.002**	0.08	0.37
	No	Ref.	Ref.	–	Ref.	Ref.

*Note*: Bold indicates statistical significance.

Abbreviations: BDT, Bangladeshi Taka; 110.16 BDT, 1 USD; CI, Confidence intervals; LL, lower limit; UL, upper limit.

^a^ Adjusted *R* ^2^: .161.

^b^ Buddhist and Christians.

Contrastingly, age, religion, administrative division, education, knowledge about nutrition, and ideas about the food safety agency of Bangladesh emerged as salient determinants of practical food label understanding (*p* < .05). Specifically, younger participants, aged between 18 and 28, showcased superior practical knowledge regarding food labels compared to their older counterparts. Younger individuals tend to be more digitally connected and knowledgeable about technology, which might give them easier access to information about nutrition and labels through online resources. It is interesting to note that participants from the Dhaka and Khulna divisions exhibited a higher level of practical knowledge of food labels. Dhaka, being the capital city, and Khulna, as an important industrial and commercial hub, are expected to have a higher level of urbanization in terms of population. Urban areas typically offer improved access to educational resources, health facilities, and a wider variety of food products. This exposure might have the potential to enhance awareness and understanding of food labeling among residents. Mymensingh, on the other hand, is recognized for its agricultural operations; however, the emphasis may be more on crop cultivation than food processing and safety, resulting in a lower level of practical knowledge of food labeling. Furthermore, educational attainment was a crucial indicator of proficiency in interpreting nutritional labels. As elucidated in Table 3, there's a positive correlation between educational levels and adeptness at label comprehension (*p* < .05). This insinuates that heightened educational endeavors are paramount in enhancing consumers' capacity to adeptly interpret food labels, especially the intricacies of nutritional data that necessitate practical expertise. Supporting this observation, Meijer et al. (2021) and Grunert et al. (2014) accentuated that education can potentiate the nutritional discernment of packaged food consumers. Similarly, Robert and Chandran (2017) identified a significant nexus between educational attainment and proficiency in label reading and nutritional comprehension.

## 4 CONCLUSION

This study underscores the centrality of food labels as pivotal tools for consumers when discerning the safety and nutritive value of packaged foods. A majority of respondents routinely scrutinize food labels, primarily owing to their perception of these labels as crucial determinants of food safety and nutrition. Specifically, respondents place a heightened emphasis on details like production and expiration dates, raw material sources, and appropriate storage and handling instructions. Consequently, they expressed a desire for more comprehensive information in these domains. Importantly, the study also revealed challenges in practical comprehension and use of nutritional information, highlighting discrepancies between perceived importance and actual understanding among consumers. Despite generally adequate perceptions of label completeness, difficulties with information clarity were identified, indicating a need for greater readability and consumer education efforts. Considering the direct correlation between education and adeptness at label comprehension, we advocate for the proliferation of educational platforms centered on food labeling in the nation, targeting both consumers and producers, to significantly enhance nutritional literacy and support more informed consumer choices. Furthermore, producers should be incentivized to refine their labeling approaches, ensuring that they are both lucid and easily interpretable.

In considering the findings of this study on consumer knowledge, perception, and practical use of food labels in Bangladesh, it is crucial to recognize certain limitations. These include the possibility of sampling bias, which may not accurately reflect the diverse Bangladeshi population. Additionally, the cross‐sectional nature of the study restricts the ability to assess changes over time, and the absence of in‐depth qualitative data hinders a comprehensive understanding of consumer motivations and challenges in relation to food labels.

Future studies should consider investigating the efficacy of educational interventions targeted at promoting food label literacy, particularly among rural and less educated groups, which may provide valuable insights. Exploring the impact of cultural and religious factors, particularly among Muslim communities, could provide insights into how dietary guidelines and cultural practices shape consumer preferences. Localized studies across various regions of Bangladesh could reveal regional disparities in label utilization, influenced by factors like access to education and food diversity. Future research could investigate particular challenges or difficulties that older individuals encounter when interpreting food label information and develop strategies to enhance their decision‐making skills and nutritional literacy. This knowledge will empower consumers to make informed food choices, ultimately contributing to improved public health outcomes.

## AUTHOR CONTRIBUTIONS

**Md. Nazrul Islam:** Conceptualization (equal); investigation (lead); methodology (equal); project administration (equal); supervision (equal); validation (lead); visualization (supporting); writing – original draft (equal); writing – review and editing (supporting). **Nitai Roy:** Conceptualization (equal); data curation (lead); formal analysis (lead); investigation (equal); methodology (supporting); project administration (equal); software (lead); supervision (equal); validation (supporting); visualization (equal); writing – original draft (equal); writing – review and editing (lead). **Felix Kwashie Madilo:** Visualization (supporting); writing – original draft (equal); writing – review and editing (supporting). **Adenike Akinsemolu:** Visualization (supporting); writing – review and editing (supporting). **Md. Arifuzzaman:** Methodology (supporting); writing – review and editing (supporting). **Md. Imran Hossain Shakil:** Methodology (supporting); writing – review and editing (supporting). **Jannatul Ferdous Nishi:** Methodology (supporting); writing – review and editing (supporting). **Sharmin Akter:** Methodology (supporting); writing – review and editing (supporting). **Md. Elius:** Methodology (supporting); writing – review and editing (supporting). **Md. Shahidul Islam:** Methodology (supporting); writing – review and editing (supporting).

## FUNDING INFORMATION

This research did not receive any specific grant from funding agencies in the public, commercial, or not‐for‐profit sectors.

## CONFLICT OF INTEREST STATEMENT

The authors declare that they have no known competing financial interests or personal relationships that could have appeared to influence the work reported in this paper.

## Data Availability

Data will be made available on request.
